# A retrospective review of the management of patients following a malignancy diagnosis on biologic therapies for the treatment of dermatological disorders

**DOI:** 10.1016/j.jdcr.2023.07.007

**Published:** 2023-07-17

**Authors:** Paula Finnegan, Kashif Ahmad, Muriel Sadlier, Maeve Lynch

**Affiliations:** Department of Dermatology, University Hospital Limerick, Dooradoyle, Limerick, Ireland

**Keywords:** biologics, general dermatology, medical dermatology, oncology

## Introduction

Biologic agents (BAs) have transformed the management of multiple immune-mediated dermatological conditions, leading to significant improvements in quality of life for conditions such as psoriasis, atopic dermatitis, and hidradenitis suppuritiva (HS).^1^ They are engineered monoclonal antibodies and fusion proteins that target specific cytokines or cytokine receptors in the inflammatory pathway.^2^ However, biologic therapies are not without risk, most commonly an increased incidence of infection.^3^ Another potential risk previously examined is that of malignancy (see Table I).^2^ This risk albeit low appears to be most prominent in patients receiving antitumor necrosis factor agents for more than 12 months.^3^ The main cancers associated with antitumor necrosis factor BAs include lymphoproliferative disorders^3^ and nonmelanomatous skin cancers (NMSCs),^23^ as previously demonstrated by Patients in the Psoriasis Longitudinal Assessment and Registry (a US-based registry of 12,090 patients).^23^^,^^24^

**Table I:** Available articles studying the association between biologic drugs in patients with psoriasis and a history of cancer

Study	Biologic agent(s)	Number of patients diagnosed with cancer	Cancer(s) diagnosed
Balato et al^4^	Etanercept Infliximab	1	Chronic lymphocytic leukemia
Bellinato et al^5^	Secukinumab Ixekizumab	10	Melanoma (3) Prostate carcinoma (2) Rectal adenocarcinoma (1) Breast adenocarcinoma (2) Multiple myeloma (1) Bladder carcinoma (1)
Blauvelt et al^6^	Guselkumab	2	Breast cancer Lung cancer
Gambardella^7^	Secukinumab	1	Bladder carcinoma
Ghazanfar et al^8^	Secukinumab Ustekinumab	1	Melanoma
Gkalpakiotis et al^9^	Etanercept Ustekinumab	1	Melanoma
Kamiya et al^10^	Guselkumab	1	Nonsmall cell lung cancer
Jin et al^11^	Ixekizumab	1	Multiple myeloma
Lasagni et al^12^	Secukinumab Ustekinumab	1	Breast adenocarcinoma
Mastorino et al^13^	Anti-TNF	4	Melanoma Breast cancer Multiple BCC Meningioma
Mastorino et al^14^	Guselkumab Etanercept Adalimumab Ustekinumab	7	Nonmelanomatous skin cancer (3) Leiomyosarcoma (1) Clear cell kidney cancer (1) Breast cancer (1) Melanoma (1)
Odorici et al^15^	Secukinumab Ustekinumab Etanercept Infliximab Adalimumab Etanercept Efalizumab	14	Melanoma (2) Prostate carcinoma (4) Bladder adenocarcinoma (2) Pulmonary adenocarcinoma (2) Rectal adenocarcinoma (2) Clear cell renal carcinoma (1) Breast adenocarcinoma (1)
Patel et al^16^	Etanercept Ustekinumab	1	Colon cancer
Pellegrini et al^17^	Secukinumab	43	Melanoma (8) Breast cancer (7) Colon cancer (7) Lung cancer (5) Prostate cancer (4) Bladder cancer (5) Kidney cancer (3) Uterine cancer (1) Hodgkin lymphoma (1) Testicular cancer (1)
Peter et al^18^	Secukinumab	1	Melanoma
Porcar Saura et al^19^	Etanercept Adalimumab Ixekizumab	1	Laryngeal squamous cell carcinoma
Valenti et al^20^	Ixekizumab Secukinumab Ustekinumab Etanercept Guselkumab Risankizumab	16	Breast cancer (4) Nonsmall cell lung cancer Lymphoma (3) Meningioma (1) Clear cell renal carcinoma (2) Bladder cancer Duodenal adenocarcinoma Endometrial cancer
van Lümig et al^21^	Alefacept Etanercept Adalimumab	4	Breast cancer
Wang et al^22^	Ustekinumab	1	Kaposi sarcoma

*BCC*, Basal cell carcinoma; *TNF*, tumor necrosis factor.

Any treatment that alters the immune system may impair immunosurveillance, and is best avoided if possible following a cancer diagnosis.^3^^,^^23^ Current guidelines from the British Association of Dermatology^25^ and EuroGuiDerm^1^ recommend a 5-year period if possible between cancer diagnosis and treatment and commencement of a BA, and also emphasize the importance of discussion with oncology.^24^ The Joint American Academy of Dermatology and National Psoriasis Foundation Guidelines^2^ point out the lack of definitive evidence between interleukin (IL)-12/IL-23 and IL-17 inhibitors and solid tumor or lymphoreticular malignancies for psoriasis treatment.^2^ However, they also mention the need for large long-term follow-up studies to fully explore the risk of malignancy. More recent studies have suggested that there is no increased risk of solid organ malignancy in patients on the newer BAs targeting IL-12, IL-23, and IL-17A.^26^ The IL-23 inhibitor guselkumab has been used in clinical trials (VOYAGE 1 and 2) in patients with a cancer history.^6^^,^^27^

In practice, managing patients with severe dermatological disease following a diagnosis of malignancy is challenging.^25^ Available evidence to guide dermatologists in the management of these patients is significantly lacking.^13^ The British Association of Dermatology guidelines recommend oncology involvement in assisting with these decisions, as well as patient education on potential treatment risks, and the need for compliance with national cancer screening programmes.^25^

## Case series

Ethical approval was granted by the University Hospital Limerick Group research ethics committee. Patients who were diagnosed with cancer while on BAs were identified from our Filemaker Pro (Claris International Inc.) computer database, and a retrospective chart review was performed. Two hundred four letters were reviewed using 63 key word search terms (see Appendix 1, available via Mendeley at https://doi.org/10.17632/mxmxcfbggg.1). The exclusion criteria were: <18 years of age, or diagnosed with a NMSC. Written informed consent was obtained. We assessed the management of these patients including: discontinuing biologic therapies, alternative agents used for their dermatological disease, whether multiple consecutive agents were required to obtain disease control, whether BAs were restarted, and malignancy outcomes.

Eleven patients with dermatological disorders were diagnosed with malignancy while on biologic therapies (see Table II). BAs were prescribed for the treatment of: psoriasis (*n* = 9), HS (*n* = 1), and dual pathology of cutaneous Crohn’s disease and HS (*n* = 1). The mean duration of biologic therapy prior to cancer diagnosis was 20.22 ± 19 months. The mean age of patients at the time of cancer diagnosis was 59.6 ± 15.2 years. The malignancies diagnosed were: lymphoma (*n* = 3), ovarian cancer (*n* = 2), breast cancer (*n* = 1), esophageal cancer (*n* = 1), gastric cancer (*n* = 1), bladder cancer (*n* = 1), testicular cancer (*n* = 1), and lentigo maligna (*n* = 1). Biologic treatment was stopped for 9 patients immediately following cancer diagnosis. Two patients were continued on BAs. One of these patients was receiving adalimumab for the treatment of genital cutaneous Crohn’s disease and HS—this was halted within 2 years of being diagnosed with testicular cancer; the second patient who continued his BA (ustekinumab) for psoriasis was diagnosed and treated for an in situ neoplasm (lentigo maligna).

**Table II:** Patient characteristics

Patient	Age (y)	Sex	Dermatology diagnosis	Biologic agent	Cancer diagnosis	Biologic stopped?	Dermatology disease status	Cancer status	1st alternative agent used	2nd alternative	3rd alternative	4th alternative	Biologic restarted?
1	55	F	Psoriasis	Adalimumab	Breast Cancer (Ca)	Y	Flared	Remission	Acitretin	UV-B	Acitretin	-	Y—after 2 y for refractory disease (PASI 18.2 and DLQI 16) (adalimumab) following specialist discussion at a regional dermatology meeting with oncology follow-up
2	54	F	Hidradenitis suppuritiva (HS)	Adalimumab	Gastric Ca	Y	Flared	Remission	Oral antibiotics	Metformin	Intralesional steroids	Topical	N
3	61	M	Psoriasis	Ustekinumab	Bladder Ca	Y	Flared	Remission	Acitretin and topical agents	-	-	-	N
4	73	M	Psoriasis	Adalimumab	LM	N	Controlled	In situ disease only	-	-	-	-	-
5	73	M	Psoriasis	Adalimumab	Lymphoma	Y	Flared	Remission	Topical agent	-	-	-	Y—after 2 y switched to secukinumab for severe psoriatic arthritis (initiated by rheumatology)
6	50	F	Psoriasis	Etanercept	Lymphoma	Y	Flared	2nd malignancy (breast Ca)	Apremilast	Acitretin and Psoralen plus UV-A (PUVA)	Methotrexate	Hydroxyurea and acitretin	N
7	25	M	Cutaneous Crohn’s disease and HS	Adalimumab	Testicular Ca	Stopped after 2 y	Flared	Relapse	Oral antibiotics (ciprofloxacin)	Oral antibiotics (rifampicin and clindamycin)	Surgery	-	N
8	71	F	Psoriasis	Adalimumab	Ovarian Ca	Y	Flared	Terminal	Emollients	-	-	-	N
9	68	M	Psoriasis	Adalimumab	Esophageal Ca	Y	Flared	Terminal	Topical agents	-	-	-	N
10	49	F	Psoriasis	Etanercept	Ovarian Ca	Y	Flared	Terminal	Phototherapy	Acitretin	Topical agents	-	N
11	77	F	Psoriasis	Etanercept	Lymphoma	Y	Flared	Terminal	Methotrexate	-	-	-	N

*DLQI*, Dermatology life quality index; *LM*, lentigo maligna; *N*, no; *PASI*, psoriasis assessment severity index; *Y*, yes.

All 9 patients who discontinued BAs flared (see Table II). Four patients with psoriasis were controlled after switching to an alternative treatment. The remaining 4 patients with psoriasis flared after the first-trial alternative agent. Four patients required a third agent. Surgical intervention in Crohn’s disease was successful. Acitretin and hydroxyurea combined were introduced as fourth-trial agents for 1 patient with psoriasis.

Biologics were recommenced in 2 cases approximately 2 years after cancer diagnosis, both due to recalcitrant psoriasis. Adalimumab was the original agent for both patients. The first patient was diagnosed with breast cancer in 2011, undergoing lumpectomy and radiotherapy in the same year. Adalimumab was held once her cancer diagnosis confirmed, but was then restarted in 2013 following unsuccessful management with 3 alternative treatments, including acitretin, phototherapy, and a further course of acitretin. Prior to recommencing her BA, her case was discussed at a regional dermatology meeting with specialist input. Before recommencing adalimumab, her psoriasis was severe (Psoriasis Assessment Severity Index [PASI] 18.2), and her Dermatology Life Quality Index was 16, indicating a significant impact on her quality of life. Within 3 months of recommencing adalimumab, her psoriasis improved (PASI = 4.2 and Dermatology Life Quality Index = 3). The second patient was diagnosed with Non-Hodgkin lymphoma in 2018, and underwent radiotherapy in the same year. Adalimumab was held at the time of the lymphoma diagnosis. He was commenced on topical agents to manage his psoriasis, and the selective cyclo-oxygenase-2 inhibitor etoricoxib by rheumatology for treatment of psoriatic arthritis. However, his arthritis flared, and he was commenced on the IL-17A inhibitor secukinumab in 2020 by rheumatology.

Remission of malignancy was achieved in 6 cases. Relapse occurred in 1 patient (testicular cancer). One patient who was originally diagnosed with cutaneous T-cell lymphoma went on to be diagnosed with another de novo malignancy (breast cancer) 5 years later—the BA (etanercept) had remained on hold during the interval between the 2 cancer diagnoses.

Four patients subsequently died as a result of their underlying malignancy, all of whom had their BA held after the malignancy diagnosis without recommencement. All 4 patients had been attending dermatology with psoriasis. The BAs the patients received until the time of malignancy diagnosis were the antitumor necrosis factor medications adalimumab (*n* = 2) and etanercept (*n* = 2). The neoplasms were: ovarian cancer (*n* = 2), esophageal cancer (*n* = 1) and lymphoma (*n* = 1) (see Table II).

## Discussion

Our findings support the complexity of managing dermatology patients who develop malignancies on biologic therapies. Multiple successive agents were often required to gain control of skin disease. While in practice we aim to avoid BAs for at least 5 years after the malignancy diagnosis consistent with current guidelines, in reality this can be challenging.

Topical therapies may be useful for limited psoriasis, but patients requiring BAs tend to have severe disease requiring systemic treatment. Six patients were treated with topical agents as alternative therapies. In combination with acitretin, topical therapies were sufficient for control of psoriasis in 1 case. For a patient with HS, topical therapy was the most recent approach to treatment, and a response is awaited. For a patient with psoriasis and psoriatic arthritis, it failed to control his cutaneous disease. Topical agents were also used for 3 patients who died from their malignancy while on this regime.

Phototherapy can be an effective option, with up to 90% clearance rates reported.^28^ However, the duration of remission is 6 months on average, and attending for phototherapy can be difficult for many patients to coordinate.^28^ Phototherapy was used for 3 of our patients—narrowband–UV-B (*n* = 2), and psoralen plus UV-A in combination with acitretin (*n* = 1). In each case, the treatment failed, and an alternative approach was needed.

Acitretin, the vitamin-A analog, is modestly effective at treating psoriasis.^28^ Response rates can vary widely and are often dose-dependent, with partial remission (75% reduction in PASI) (PASI-75) being achieved in 25% to 75% of patients.^28^ Four patients were treated with acitretin. It successfully resulted in control of psoriasis when used in combination with hydroxyurea in 1 case. In the other 3 patients, it failed to induce remission.

Methotrexate, an immunosuppressive agent, has been used for psoriasis since 1958.^28^ Research has shown it can achieve a response rate of PASI-75 in 37.5% of patients after 16 weeks of treatment.^29^ Methotrexate is not a promoter or inducer of cancer, and is often used as a chemotherapeutic agent for solid organ tumors.^30^ Registry data have been reassuring to date, with no increased risk of malignancy seen with low-dose methotrexate exposure in psoriasis patients, regardless of duration of treatment.^23^ In a recent Inference-Based Guidance from a Multidisciplinary Expert Panel, Papp et al concluded that in psoriasis patients with a history of solid organ malignancy, methotrexate exposure is unlikely to alter prognosis related to their previous malignancy.^30^ Methotrexate was trialed as an alternative treatment for 2 patients. It failed to control disease in 1 patient. The second patient died from their malignancy while on this regimen.

While there have been no randomized controlled trials looking at the efficacy of hydroxyurea in the treatment of psoriasis, it has been shown to be a modestly effective therapy.^31^ Originally a systemic anticancer therapy, it is not contraindicated in patients with malignancy, and may theoretically reduce the risk of NMSCs.^31^ Hydroxyurea was used in combination with acitretin for one case of psoriasis—the outcome is awaited.

The Efficacy and Safety Trial Evaluating the Effects of Apremilast in Psoriasis-1 trial assessed the efficacy of apremilast, a phosphodiesterase inhibitor licensed for psoriasis and psoriatic arthritis.^32^^,^^33^ It demonstrated a moderate response rate, with a PASI-75 of 33% after 16 weeks of treatment.^32^^,^^33^ Di Lernia et al carried out a retrospective study assessing apremilast for the management of psoriasis in patients with a malignancy history.^34^ They identified 14 patients, 8 of whom continued apremilast for a duration of at least 36 months. None of those 8 patients developed relapses of their malignancy. Of the remaining 6 patients, 2 of those developed recurrence of their malignancy, but they had discontinued apremilast within 5 months due to inefficacy or adverse effects. In our series, 1 patient with psoriasis was treated with apremilast, but it failed to control the patient’s skin disease.

There is a paucity of data on the safety of BAs in patients who have a history of malignancy. These patients are typically excluded from clinical trials. Table I outlines studies and case reports/series available in the literature focusing on patients diagnosed with previous malignancy who received a BA for the treatment of psoriasis. Mastorino et al^13^ reported a large series of 38 real-world patients with psoriasis with a history of cancer who were subsequently treated with a BA. They found biologic treatments to be safe in this special population.^13^ Blauvelt et al conducted the VOYAGE 1 and 2 trials—these were randomized double-blinded placebo/active comparator-controlled trials that compared the novel IL-23 agent guselkumab with placebo and adalimumab. Patients recruited included those with a cancer history with no recurrence for more than 5 years before screening.^6^ Out of 1826 patients, they identified 20 who had a history of malignancy. Of those 20 who were exposed to guselkumab, there were just 2 malignancies reported. One was a new primary malignancy—metastatic breast cancer was diagnosed in a patient with a history of previous prostate cancer, leading to study withdrawal. The second involved recurrence of their primary malignancy (lung cancer), which was fatal. NMSCs were reported in 2 patients. Blauvelt et al outline the need for further investigation with larger sample sizes over a longer follow-up duration to allow a better understanding of the future malignancy risk of BAs for dermatology patients with a cancer history.^6^

In conclusion, we report the dermatological management of 11 patients who developed cancers during treatment with BAs. Where BAs were discontinued, all patients developed flares of their dermatological disease, highlighting the significant clinical challenge of managing these patients. Many patients required multiple consecutive agents in an effort to control their disease. In 2 patients, the decision was made to restart biologic treatment when other measures failed. Limitations of our study included a small sample size. Patient education is a vital part of managing this special population. Longstanding pharmacovigilance is needed in dermatology patients requiring systemic treatment who develop malignancy and subsequently require treatment with BAs for clarification of patient safety and long-term adverse effects.

## Conflicts of interest

None disclosed.
